# Effectiveness and Safety of Left Distal Transradial Access in Coronary Procedures in the Caribbean

**DOI:** 10.7759/cureus.54601

**Published:** 2024-02-21

**Authors:** Naveen A Seecheran, Abel Y Leyva Quert, Valmiki K Seecheran, Rajeev V Seecheran, Arun Katwaroo, Cathy-Lee Jagdeo, Salma Rafeeq, Priya Ramcharan, Lakshmipathi Peram, Ravi Ramlal, Anil Ramlackhansingh, Stanley Giddings, Sherry Sandy

**Affiliations:** 1 Clinical Medical Sciences, The University of the West Indies, St. Augustine, TTO; 2 Cardiology, Eric Williams Medical Sciences Complex, Mt. Hope, TTO; 3 Internal Medicine, Eric Williams Medical Sciences Complex, Mt. Hope, TTO; 4 Internal Medicine, University of Kansas Medical Center, Wichita, USA; 5 Internal Medicine, Trinidad Institute of Medical Technology, St. Augustine, TTO

**Keywords:** primary percutaneous coronary intervention (pci), radial artery occlusion (rao), snuffbox access, distal transradial access (dtra), transradial access (tra)

## Abstract

Introduction: This retrospective study investigated the effectiveness and safety of left distal transradial access (LDTRA) in patients with cardiovascular disease in Trinidad undergoing coronary angiography (CAG) or percutaneous coronary intervention (PCI).

Method: Procedural parameters, including technical success and safety outcomes such as vascular complications and radial artery occlusion (RAO), were assessed in 111 consecutive patients undergoing CAG or PCI from January 2023 to June 2023 at the Eric Williams Medical Sciences Complex, Trinidad and Tobago. Eighty-eight patients underwent LDTRA, while 23 received left transradial access (LTRA).

Results: There was no difference in procedural success with LDTRA compared to LTRA, 90.9% vs. 100%, *p*-value 0.202, non-significant (ns). LDTRA was associated with shorter fluoroscopy times (8.4 ± 6.8 minutes vs. 12.4 ± 7.7 minutes, *p*-value = 0.02), procedural duration (26.7 ± 18 minutes vs. 35.8 ± 20 minutes, *p*-value = 0.04), and hemostasis time (142 ± 41 minutes vs. 186 ± 44 minutes, *p*-value < 0.05). There were no significant differences in procedural-related complications (8% for LDTRA vs. 4.3% for LTRA, *p*-value = 0.476, ns). There were no reported cases of RAO. In the subgroup of patients with prior coronary artery bypass grafting (CABG), the fluoroscopy and procedure times were similar for both access sites; however, LDTRA was associated with a shorter hemostasis time (128 ± 30 minutes vs. 194 ± 39 minutes, *p*-value = 0.01).

Conclusions: LDTRA is effective and safe for coronary procedures and is associated with a shorter hemostasis time. This study may prove clinically pertinent in a limited-resource Caribbean setting.

## Introduction

Transradial access (TRA) has almost entirely supplanted transfemoral access (TFA) as the primary vascular access site for coronary procedures. This preferred route has been firmly incorporated within the European Society of Cardiology/European Association for Cardio-Thoracic Surgery (ESC/EACTS) and American College of Cardiology/American Heart Association/Society for Cardiovascular Angiography and Interventions (ACC/AHA/SCAI) guidelines for coronary angiography (CAG) and percutaneous coronary intervention (PCI), as evidenced by several high-fidelity meta-analyses and systematic reviews [1-3].

The effectiveness and safety of right (R) and left (L) TRA were compared in a now decade-old randomized left versus right transradial approach for percutaneous coronary procedures (TALENT) trial, which revealed significantly shorter fluoroscopy times with left transradial access (LTRA), which was attributed to a three-fold higher incidence of subclavian tortuosity and a higher incidence of radial loops associated with RTRA [4]. Procedural success rate, overall duration, number of catheters, and volume of contrast media used were similar [4]. A comparable trend was also observed in patients undergoing TRA for acute coronary syndromes (ACS) and alluded to no significant difference between RTRA and LTRA with respect to procedural success, duration, room-to-balloon time, and safety profile [5]. A large Chinese meta-analysis evaluating over 10,000 patients demonstrated that LTRA was superior to RTRA in terms of fluoroscopy time and contrast media volume for diagnostic and interventional coronary procedures [6].

Distal radial access (DRA) in the anatomical snuff box or the dorsum of the hand has emerged as a novel access site to attenuate the risk of radial artery occlusion (RAO) due to the anastomotic network, which continuously perfuses the radial artery [7]. Two recent randomized clinical trials (RCTs) displayed a marked reduction of RAO after DRA compared with conventional TRA [8].

This retrospective study investigated the effectiveness and safety of left distal transradial access (LDTRA) in coronary procedures at a tertiary center in Trinidad and Tobago.

## Materials and methods

**Figure 1:**
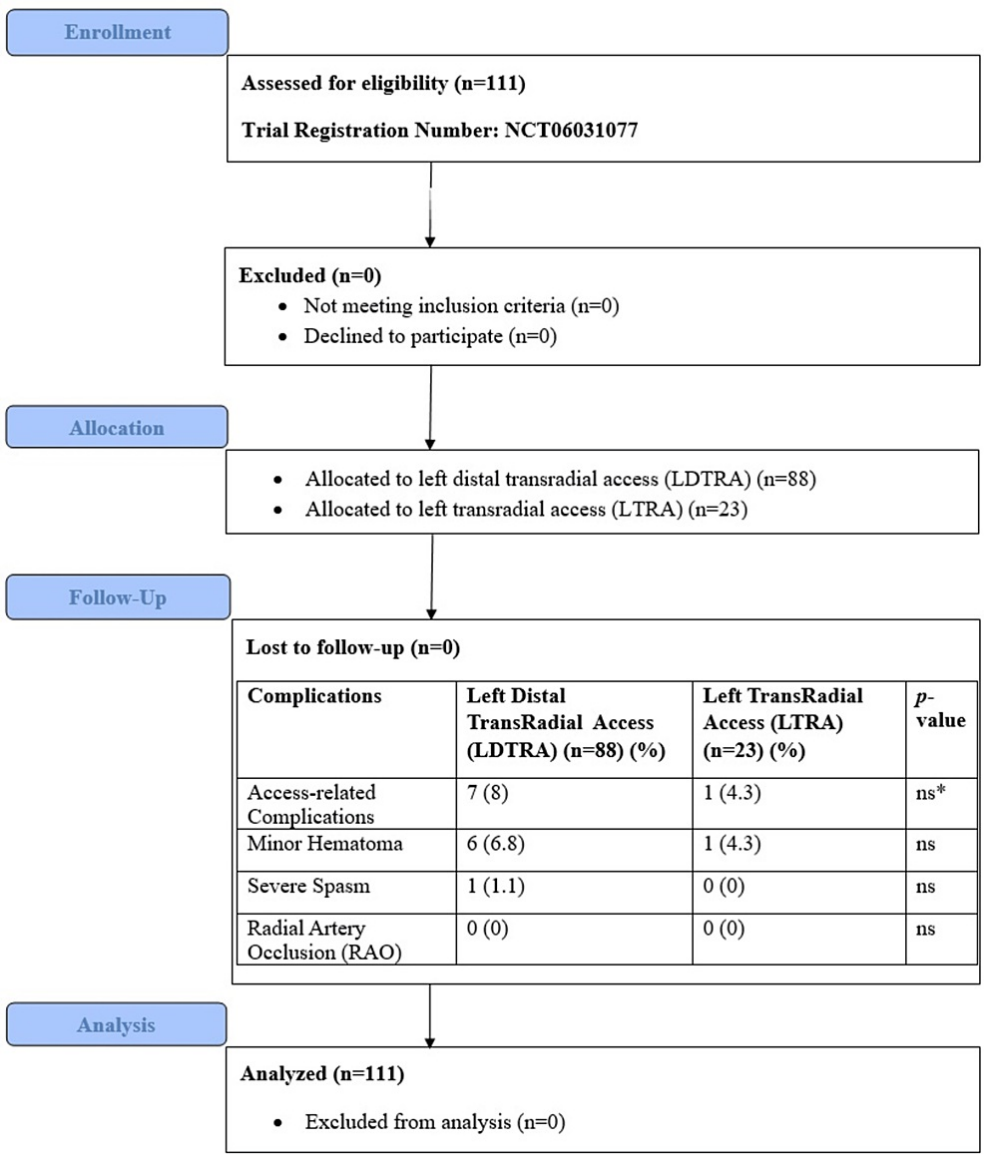
Consolidated standard of reporting trials (CONSORT) flow diagram. *Fisher′s exact test; ns: non-significant.

The study complied with the Declaration of Helsinki and the International Conference on Harmonization and Good Clinical Practice and was approved by the Campus Research Ethics Committee (CREC) of the University of the West Indies, St. Augustine (UWI STA), Trinidad (CREC-SA.1304/12/2021). This trial was registered on the ClinicalTrials.gov site, with identification NCT06031077. The retrospective study analyzed patients presenting to the Cardiac Catheterization Laboratory (Allura Xper FD20, Philips Healthcare, Amsterdam, Netherlands) at the Eric Williams Medical Sciences Complex (EWMSC), Trinidad and Tobago, an academic medical center for either CAG or PCI, between January 2023 and June 2023. The inclusion criteria comprised patients with any Consultant Cardiologist-approved indication for CAG, also verified by the Consultant Interventional Cardiologist. Patients were followed up 24 hours and one month post-procedurally. RAO was assessed with a modified reverse Barbeau’s test (MRBT) with a Spacelabs Ultraview SL 2400 monitor (Spacelabs Healthcare, Snoqualmie, Washington, United States) (Figure 1) [3].

Data were collected via case report forms by an interventional cardiology registrar (who completed a one-year interventional cardiology fellowship in Galicia, Spain) and a clinical research associate assessing demographic data, which included the patient’s medical and procedural history. This study personnel did not participate in actual CAGs and PCIs; however, they entered the data into the case report forms and electronic database, ensuring a case-control, single-blinded design. CAGs and PCIs were performed by the American Board of Internal Medicine (ABIM)-certified Consultant Interventional Cardiologist (also a Fellow of the Society of Cardiac Angiography and Interventions [FSCAI]), who has performed TRA procedures for at least a decade. The radial sheaths utilized were five French or six French Cordis AVANTI®+ Introducers (Cordis®, Santa Clara, California, United States). The intraarterial radial “cocktail” for CAG comprised 5000 units of heparin and 250 µg of nitroglycerin, while PCI included 70 units per kilogram (weight-based adjusted dosing with a minimum of 5000 units) and 250 µg of nitroglycerin (verapamil was not routinely available in our setting). TR BAND® Radial Compression Device (Terumo Corporation, Tokyo, Japan) was utilized post-procedure for a minimum of two hours for both DRA and TRA [9-10]. The Cardiac Catheterization Laboratory certified the nursing personnel and technical assistants. Incentives were not offered for participating in the study. All data were secured in the Department of Clinical Medical Sciences (DCMS) office at the EWMSC, UWI STA, to maintain confidentiality. Computer data were accessible only to the biostatistician, password-protected, and database-encrypted. During the data collection and analysis, no patient identifiers were included.

Statistical analysis

Based on a quantitative study, we calculated a sample size of 88 patients to detect an association, with an alpha (α) value of 0.05, a power of 80%, and an absolute delta Δ of 25% reduction in RAO with LDTRA compared to LTRA. Statistical analysis was performed using SPSS® 28.0.1 (IBM, New York City, New York, USA). The parameters and outcomes were investigated using descriptive statistics. Percentages represented categorical data, and means with standard deviations (SDs) were used for continuous data. The comparisons between samples for categorical data used Chi-squared tests; however, comparisons between groups for continuous data were made using students’ t-tests and contingency tables using Fisher’s test. All p-values were two-tailed; any value < 0.05 was considered statistically significant.

## Results

The average age of the participants was almost 60 years (59.9 ± 10 years), with nearly one-third being female. Appropriate indications for CAG and PCI included chronic coronary syndromes (CCS) (34-52%) and ST-segment-elevation acute coronary syndrome (STE-ACS) (17.4-30.7%). Approximately one-quarter of patients had heart failure with reduced ejection fraction (HFrEF), with more than one-half to three-quarters having diabetes mellitus (DM). There was a statistically significant disparity in patients with prior coronary artery bypass surgery (CABG), with a higher percentage assigned to LTRA (43.5% vs. 15.9%, p-value 0.009; Table 1).

**Table 1:** Patient demographics. *Fisher′s exact test; **student’s t-test; ns: non-significant. LDRA: left distal transradial access, LTRA: left transradial access, CCS: chronic coronary syndrome, STE-ACS: ST-segment-elevation acute coronary syndrome, NSTE-ACS: non-ST-segment-elevation acute coronary syndrome, USA: unstable angina, AS: aortic stenosis, CABG: coronary artery bypass grafting, PCI: percutaneous coronary intervention, ACS: acute coronary syndrome, HFrEF: heart failure with reduced ejection fraction, LVEF: left ventricular ejection fraction, DM: diabetes mellitus, HTN: hypertension, HLD: dyslipidemia, CKD: chronic kidney disease, PAD: peripheral arterial disease.

Variables	LDTRA (n=88) (%)	LTRA (n=23) (%)	p-value
Age (mean ± standard deviation) (years)	59.9 ± 10	59.2 ± 11	0.798, ns**
Height (mean ± standard deviation) (m)	1.67 ± 0.1	1.68 ± 0.1	0.831 ns**
Weight (mean ± standard deviation) (kg)	75 ± 18	83.5 ± 23	0.058, ns**
Female gender	27 (30.7)	7 (30.4)	0.982, ns
South Asian ethnicity	70 (80)	17 (74)	0.443, ns
Clinical presentation			
CCS	30 (34.1)	12 (52.2)	0.11, ns
STE-ACS	27 (30.7)	4 (17.4)	0.20, ns
NSTE-ACS	16 (18.2)	3 (13)	0.75, ns*
USA	10 (11.4)	3 (13)	0.73, ns*
AS	2 (2.3)	1 (4.3)	0.27, ns
Other	3 (3.4)	0 (0)	0.62, ns
Procedural history			
Prior CABG	14 (15.9)	10 (43.5)	*0.009
Prior PCI	8 (9.1)	3 (13)	0.695, ns*
Comorbidities			
Prior ACS	14 (15.9)	2 (8.7)	0.516, ns*
HFrEF with LVEF < 40%	20 (22.9)	6 (26.1)	0.735, ns
DM	53 (60.2)	17 (73.9)	0.226, ns
HTN	68 (77.3)	15 (65.2)	0.236, ns
HLD	22 (25)	8 (34.8)	0.347, ns
Chronic tobacco use	22 (25)	5 (21.7)	0.746, ns
CKD	6 (6.8)	3 (13)	0.390, ns*
PAD	4 (4.5)	1 (4.3)	0.99, ns*
Obesity	18 (20.5)	8 (34.8)	0.149, ns

There was no difference in procedural success with LDTRA compared to LTRA, 90.9% vs. 100%, p-value = 0.202, non-significant (ns). LDTRA was associated with shorter fluoroscopy times (8.4 ± 6.8 minutes vs. 12.4 ± 7.7 minutes, p-value = 0.02), procedural duration (26.7 ± 18 minutes vs. 35.8 ± 20 minutes, p-value = 0.04), and hemostasis time (142 ± 41 minutes vs. 186 ± 44 minutes, p-value = 0.01) (Table 2). There were no significant differences in procedural-related complications (8% for LDTRA vs. 4.3% for LTRA, p-value = 0.476). There were no reported cases of RAO (Table 3). In the subgroup of patients with prior CABG, the fluoroscopy and procedure times were similar for both accesses; however, the LDTRA was associated with a shorter hemostasis time (128 ± 30 minutes vs. 194 ± 39 minutes, p-value = 0.01) (Table 4).

**Table 2:** Comparison of left distal transradial access versus left transradial access. *Fisher′s exact test; **student’s t-test; ns: non-significant. LDRA: left distal transradial access, LTRA: left transradial access, PCI: percutaneous coronary intervention, CAG: coronary angiography.

Variables	LDTRA (n=88) (%)	LTRA (n=23) (%)	p-value
Procedures			
CAG	71 (80.7)	20 (87)	0.761, ns*
Urgent PCI	12 (13.6)	1 (4.3)	0.296, ns*
Elective PCI	5 (5.7)	2 (8.7)	0.633, ns*
Number of catheters used (mean ± standard deviation)	1.6 ± 0.7	2 ± 0.9	0.006**
6 French arterial sheath	44 (50)	17 (73.9)	0.04
Need for arterial access crossover	8 (9.1)	0 (0)	0.202, ns*
Successful procedure	80 (90.9)	23 (100)	0.202, ns*
Fluoroscopy time (mean ± standard deviation) (minutes)	8.4 ± 6.8	12.4 ± 7.7	0.02**
Procedural time (mean ± standard deviation) (minutes)	26.7 ± 18	35.8 ± 20	0.04**
Hemostasis time (mean ± standard deviation) (minutes)	142 ± 41	186 ± 44	0.01**

**Table 3:** Procedural complications of patients who underwent left distal transradial access versus left transradial access. *Fisher′s exact test; ns: non-significant. LDRA: left distal transradial access, LTRA: left transradial access, RAO: radial artery occlusion.

Complications	LDTRA (n=88) (%)	LTRA (n=23) (%)	p-value
Access-related complications	7 (8)	1 (4.3)	0.476, ns*
Minor hematoma	6 (6.8)	1 (4.3)	0.218, ns
Severe spasm	1 (1.1)	0 (0)	0.639, ns
RAO	0 (0)	0 (0)	0.415, ns

**Table 4:** Procedural complications of patients who underwent left distal transradial access versus left transradial access in the coronary artery bypass grafting subgroup. **Student’s t-test; ns: non-significant. LDRA: left distal transradial access, LTRA: left transradial access.

Complications	LDTRA (n=14)	LTRA (n=10)	p-value
Number of catheters used (mean ± standard deviation)	2.3 ± 0.7	2.8 ± 0.8	0.113, ns**
Fluoroscopy (mean ± standard deviation) (minutes)	13.7 ± 6	15.9 ± 7	0.415, ns**
Procedural time (mean ± standard deviation) (minutes)	34 ± 13	41 ± 11	0.174, ns**
Hemostasis time (mean ± standard deviation) (minutes)	128 ± 30	194 ± 39	0.01**

## Discussion

TRA benefits comprise decreased mortality despite complex interventional procedures [11-12]. It is also linked to superior quality of health- and economy-related metrics, thus rapidly gaining traction as the initial favored approach for CAG and PCI and other interventional procedures performed by specialties such as radiology and neurology [12-13]. Recently, CAG and PCI via the DTRA have demonstrated advantages attributed to less access to site-related complications, notably RAO with a shorter hemostasis duration than conventional TRA [14-15]. Moreover, the feasibility of the DTRA for patients with CAD, including ST-segment-elevation acute coronary syndrome myocardial infarction (STE-ACS), has also replicated fewer complications [16-18]. There is still inertia with respect to the adoption of this novel technique, despite its benefits, due to the paucity of data concerning the learning curve, such as the number of cases required to achieve a high proficiency level; however, in some countries, all aspects of DTRA are performed by nursing personnel [15,19-20]. DRA in the anatomical snuff box has appeared as a possible successor to further reduce the incidence of RAO due to anastomotic perfusion within the hand [7,21]. An occluded radial artery may preclude its use as an arterial conduit for future surgical procedures, which have demonstrated superior patency rates [10,22-23].

The DISCO RADIAL trial was a large, multicentric, randomized controlled trial (RCT) that assessed the efficacy and safety of DRA versus conventional TRA. This was performed on the heels of two studies, each flawed by their single-center design and relatively high rates of RAO in the conventional TRA arm [8,24]. The DAPRAO (distal radial approach to prevent radial artery occlusion) and ANGIE (anatomical snuffbox for coronary angiography and interventions) studies were two RCTs comparing DRA with TRA while assessing RAO as the primary endpoint [8,24]. These studies both demonstrated relatively high rates of RAO: 8.8% and 7.9% with conventional TRA and 1.2% and 3.7% with DRA, respectively, considerably higher than the RAO rates in DISCO RADIAL. DISCO RADIAL involved 1307 patients and demonstrated that the RAO difference was not statistically significant between DRA and TRA (0.31% vs. 0.91%; p-value = 0.29). Crossover rates were higher with DRA (7.4% vs. 3.5%; p-value = 0.002), while median hemostasis time was significantly shorter (153 minutes vs. 180 minutes; p-value < 0.001). Overall, bleeding events and vascular complications did not differ between groups. The authors concluded that TRA remained the gold-standard access concordant with best-practice guidelines; however, DRA emerged as a valid alternative [25]. Our study also demonstrated directionally consistent results. There was no difference in procedural success with LDTRA compared to LTRA, 90.9% vs. 100%, p-value = 0.202, non-significant (ns). There were no significant differences in procedural-related complications (8% for LDTRA vs. 4.3% for LTRA, p-value = 0.476, ns) and no reported cases of RAO. LDTRA was associated with shorter fluoroscopy times (8.4 ± 6.8 minutes vs. 12.4 ± 7.7 minutes, p-value = 0.02), procedural duration (26.7 ± 18 minutes vs. 35.8 ± 20 minutes, p-value = 0.04), and hemostasis time (142 ± 41 minutes vs. 186 ± 44 minutes, p-value = 0.01). In a subgroup analysis of patients with prior CABG, the fluoroscopy and procedure times were similar for both accesses; however, LDTRA was associated with a shorter hemostasis time (128 ± 30 minutes vs. 194 ± 39 minutes, p-value = 0.01). There are documented differences with respect to left and right accesses (LTRA and RTRA) in terms of procedural time, but none between LTRA and LDTRA. We postulate that the distal access is approximately 2-3 inches further, which provides a more stable platform for diagnostic and guide stability for maneuvering and engaging catheters. This is an interesting viewpoint, as in the CABG subgroup, there were no differences with respect to these parameters due to the possibility of requiring more catheters to cannulate bypass grafts.

Generally, outcomes are near-equivalent when comparing RTRA and LTRA when performed by expert transradialists. The TALENT trial comprised 1,500 randomized participants and demonstrated that LTRA was associated with a significantly shorter learning curve and decreased fluoroscopy times as the operator volume increased compared to RTRA [4]. Our study displayed similarity when comparing LDTRA to LTRA with respect to fluoroscopy, procedural, and hemostatic times (8.4 ± 6.8 vs. 12.4 ± 7.7 minutes; p-value = 0.02; 26.7 ± 18 vs. 35.8 ± 20 minutes; p-value = 0.04; and 142 ± 41 vs. 186 ± 44 minutes, p-value = 0.01). With the implementation of a rigorous hemostasis protocol, DRA and TRA have equally low RAO rates. Although the DISCO RADIAL trial recorded a higher crossover rate, albeit with a shorter hemostasis time, our study did not indicate a statistically significant degree of crossover (8 [9.1%] vs. 0 [0%], p-value = 0.202), but depicted the latter [25]. This study also demonstrated that LDTRA required fewer catheters than LTRA (1.6 ± 0.7 vs. 2 ± 0.9 catheters, p-value = 0.006). Again, we theorize that this approach provides more tactile maneuverability, and thus, fewer catheters may be utilized.

RAO typically ranges between <1% and 33% and differs when assessed (7.7% at 24 hours and 5.5% at >1-week follow-up with spontaneous recanalization in 50% of patients at 30 days). Its pathophysiology is complex and multifactorial, resulting in thrombosis of the relatively small-caliber artery. There are both patient- and procedural-related risk factors for RAO, including coronary artery disease, diabetes, peripheral artery disease, chronic kidney disease, gender, radial artery diameter, body mass index, sheath size, anticoagulation, and procedural and hemostasis time [26]. The most accurate prognosticator for reducing RAO is heparin dosage, with lower doses associated with increased risk (risk ratio 0.36, 95% CI: 0.17-0.76) [27]. A Chinese study reported the incidence of RAO at 3.8% for DTRA and 12.7% for TRA. Radial artery diameter was a predictive factor for DTRAO [28]. In Indian populations, RAO was encountered in approximately 12-18% of patients undergoing coronary procedures, attributed to a smaller body surface area and radial artery diameter [29]. As a result of the adoption of RAO mitigation strategies, rates were reduced to <3% in landmark trials performed at highly experienced radial centers [30-31]. In the PROPHET-II (Prophylactic Hyperperfusion Evaluation Trial), TRA was performed with counterpuncture technique, hydrophilic sheaths, intraarterial NTG, verapamil, and heparin following cannulation, and patent hemostasis with ulnar compression, suggesting that relatively low RAO rates (<1%) are possible when adhering to best TRA practices [32-33]. There was stringent adherence to the international consensus document to mitigate RAO post-transcatheter procedures to achieve an institutional rate of early RAO < 5% [10]. Our study did not record any RAOs. The introducer sheaths were either 5 French or 6 French Cordis AVANTI®+ Introducer 11 cm (Cordis, Santa Clara, California, USA). The intraarterial radial “cocktail” for CAG comprised 5000 units of heparin and 250 micrograms of nitroglycerin, while for PCI it included 70 units per kilogram (weight-based adjusted dosing with a minimum of 5000 units) and 250 µg of nitroglycerin (verapamil was not routinely available in our setting). TR BAND® Radial Compression Device (Terumo Corporation, Tokyo, Japan) was utilized post-procedure for a minimum of two hours for both DRA and TRA. Focusing on RAO mitigation is now considered a key strategy in centers with a successful TRA program.

Our results are unique for the Caribbean, a diverse, multi-ethnic region, and provide an overview of the adoption of novel vascular access techniques such as conventional TRA compared to DRA in such a limited-resource setting. While it is notable that DRA may be challenging due to difficult anatomy with respect to the caliber of the artery and a compact snuffbox and may be facilitated by ultrasound guidance, the upside includes relatively lower RAO with timely hemostasis and equivalent procedural success.

Study limitations

This study has several limitations. First, this is a single-center study based at the national cardiac catheterization laboratory (CCL) (EWMSC) of an academic medical center (UWI STA) in Trinidad. Although the study has been adequately powered and represents the largest TRA study in the English-speaking Caribbean, these findings may not be externally valid for other populations, such as different ethnic subgroups or a geriatric patient panel.

Observer and reporting bias could potentially occur despite the Cardiology Registrar, CRA, and biostatistician quality-checking and ensuring all aspects of data collection and input from CRF to database entry. This study was single-blinded, as the interventionalist did not record any study outcomes as these tasks were assigned to other independent team members. This study was also case-controlled in an almost 4:1 ratio of LDTRA to LTRA, which would have only increased the results’ power, reliability, and accuracy.

Compared to others performed by this group, this study alluded to a high prevalence of T2DM, which is inextricably linked to negative remodeling, potentially resulting in a smaller caliber vasculature [34]. We did not record pre-procedural cardiovascular medications, such as antithrombotic therapies and vasodilators, which may possibly impact RAO [35-36]. Additionally, it has been demonstrated that Trinidadian South Asian patients have accentuated platelet reactivity and a smaller caliber of coronary arteries, both for pathophysiological and anatomical reasons that could plausibly contribute to RAO [37-38]. The majority of TRA studies evaluating outcomes predominantly involve Caucasian patients, of which there may be ethnic-related differences in patient factors. Other procedural aspects, such as radiation exposure metrics and contrast media volume, were not recorded. Despite these potential mechanisms for RAO, our study did not record any events. This may have been attributed to novel sheath introducers, adequate anticoagulation with unfractionated heparin, or possibly ethnicity-related anthropometric differences. A point-of-care vascular ultrasound was not utilized to measure radial artery dimensions, as this device is not routinely available in the CCL.

In Trinidad and Tobago’s public healthcare sector, there are multisystemic issues in delivering a clinically effective and safe service for cardiovascular patients. For example, logistically, the CCL is only available during the workday as there is insufficient staff to operate a “full-time, on-call” interventional suite [39]. Additionally, blood transfusion products are often insufficient or delayed, a critical resource for patients with severe bleeding episodes such as retroperitoneal hematoma associated with TFA. With respect to specialist personnel, there is only one Cardiothoracic and Vascular Surgery team, and they are not always accessible in cases of an emergency such as coronary artery dissection, stent embolization, severe forearm hematoma, or compartment syndrome. A complete armamentarium with a complete toolkit, including multimodality imaging such as intravascular ultrasound (IVUS) and optical coherence tomography (OCT), is unavailable in our setting. As such, a safety-first approach is paramount, as bail-out strategies are very limited [39].

Doppler ultrasonography (DUSG) is widely considered the gold standard for assessing RAO following TRA; however, digital plethysmography, using a modified reverse Barbeau’s test (MRBT), is more affordable and less time-consuming [40-41]. Although MBRT has demonstrated near-equivalency to DUSG in recent RCTs, operator error using the former assessment technique may have led to misinterpretation of RAO, for example, categorizing a true RAO as patent [42-43]. The laser Doppler scan is a novel, non-invasive method that may allow rapid diagnosis of RAO; however, this is not available in our setting [26,41,44]. RAO was assessed with a MRBT [45]. There was no pilot study, and there was no lead-in period for attempting the DTRA. The procedures for the study were performed immediately after reviewing an online video tutorial detailing the technique for access [46].

## Conclusions

Left-distal transradial access is effective, safe for coronary procedures, and associated with a shorter hemostasis time. This study may prove clinically pertinent in a limited-resource Caribbean setting. Large, multicenter, randomized, controlled trials are required to confirm these exploratory findings.
